# Role of Endoplasmic Reticulum Stress in α-TEA Mediated TRAIL/DR5 Death Receptor Dependent Apoptosis

**DOI:** 10.1371/journal.pone.0011865

**Published:** 2010-07-29

**Authors:** Richa Tiwary, Weiping Yu, Jing Li, Sook-Kyung Park, Bob G. Sanders, Kimberly Kline

**Affiliations:** 1 School of Biological Sciences/C0900, University of Texas at Austin, Austin, Texas, United States of America; 2 Department of Nutritional Sciences/A2703, University of Texas at Austin, Austin, Texas, United States of America; Roswell Park Cancer Institute, United States of America

## Abstract

**Background:**

α-TEA (RRR-α-tocopherol ether-linked acetic acid analog), a derivative of RRR-α-tocopherol (vitamin E) exhibits anticancer actions *in vitro* and *in vivo* in variety of cancer types. The objective of this study was to obtain additional insights into the mechanisms involved in α-TEA induced apoptosis in human breast cancer cells.

**Methodology/Principal Findings:**

α-TEA induces endoplasmic reticulum (ER) stress as indicated by increased expression of CCAAT/enhancer binding protein homologous protein (CHOP) as well as by enhanced expression or activation of specific markers of ER stress such as glucose regulated protein (GRP78), phosphorylated alpha subunit of eukaryotic initiation factor 2 (peIF-2α), and spliced XBP-1 mRNA. Knockdown studies using siRNAs to TRAIL, DR5, JNK and CHOP as well as chemical inhibitors of ER stress and caspase-8 showed that: i) α-TEA activation of DR5/caspase-8 induces an ER stress mediated JNK/CHOP/DR5 positive amplification loop; ii) α-TEA downregulation of c-FLIP (L) protein levels is mediated by JNK/CHOP/DR5 loop via a JNK dependent Itch E3 ligase ubiquitination that further serves to enhance the JNK/CHOP/DR5 amplification loop by preventing c-FLIP's inhibition of caspase-8; and (iii) α-TEA downregulation of Bcl-2 is mediated by the ER stress dependent JNK/CHOP/DR5 signaling.

**Conclusion:**

Taken together, ER stress plays an important role in α-TEA induced apoptosis by enhancing DR5/caspase-8 pro-apoptotic signaling and suppressing anti-apoptotic factors c-FLIP and Bcl-2 via ER stress mediated JNK/CHOP/DR5/caspase-8 signaling.

## Introduction

Targeting cell surface death receptors, especially tumor necrosis factor-related apoptosis-inducing ligand (TRAIL/Apo2L) binding receptors, holds promise for cancer treatment [1], [2]. TRAIL selectively induces apoptosis in a wide variety of cancer cells with little or no toxicity towards normal cells [1], [2]. Thus, agents that can enhance TRAIL death receptor (TRAIL-R/DR4 or TRAIL-R2/DR5) signaling or sensitize TRAIL resistant cells to TRAIL induced apoptosis are of interest [2], [3]. TRAIL/DR4/DR5 apoptotic signaling includes: interaction of TRAIL with DR4 or DR5, receptor clustering, recruitment of the adaptor molecule FADD, and activation of initiator caspases-8 or -10, leading to cleavage of downstream effector caspases (mitochondrial-independent apoptosis) or cleavage of Bid, a pro-apoptotic Bcl-2 family member, leading to mitochondrial-dependent apoptosis [4].

Evading apoptosis is a hallmark of cancer [5]. One way tumor cells can escape death signals is by expression of anti-apoptotic pro-survival proteins [6]. Therefore, targeting anti-apoptotic proteins also holds promise for killing cancer cells and sensitizing them to different therapeutics [7]. c-FLIP (cellular FADD-like IL-1α-converting enzyme inhibitory protein), is a death effector domain containing protein that regulates extrinsic death receptor signaling from the tumor necrosis factor-α (TNF-α) family of cell surface death receptors, including DR4/DR5, Fas (CD95/APO-1), and TNFR [8]. c-FLIP is a catalytically inactive caspase-8/10 homolog and typically functions as a caspase-8 inhibitor resulting in chemotherapeutic drug resistance [8].

α-TEA [2,5,7,8-tetramethyl-2R-(4R,8R,12-trimethyltridecyl)chroman-6-yloxyacetic acid], called RRR-α-tocopherol ether-linked acetic acid analog or RRR-α-tocopheryloxyacetic acid is a nonhydrolyzable ether analog of RRR-α-tocopherol [9]. α-TEA has been shown to be a potent pro-apoptotic agent both in *vitro* and *in vivo* in breast, prostate and ovarian cancer cells [10], [11], [12], [13], [14], [15]. Recently, α-TEA has been shown to delay tumor onset and inhibit the progression and metastatic spread in a clinically relevant model of spontaneous breast cancer, further highlighting the translational potential of this anticancer agent [16]. Mechanisms involved in α-TEA induced apoptosis include: activation of JNK/c-Jun, p73/NOXA and Fas, as well as suppression of c-FLIP-L, survivin and phospho-Akt (pAkt), leading to death receptor mediated caspase-8 activation and mitochondria dependent apoptosis [17], [18], [19], [20], [21], [22], [23].

These data are the first to show that α-TEA induces ER stress dependent increases in death mediators JNK/CHOP/DR5 and decreases in survival mediators c-FLIP-L and Bcl-2 in human breast cancer cells. These ER stress mediated events function downstream of α-TEA triggered TRAIL/DR5/caspase-8 signaling, leading to up-regulation of JNK, CHOP and DR5 and downregulation of c-FLIP and Bcl-2.

## Materials and Methods

### Chemicals

α-TEA was made in house as previously described [9]. ER stress inhibitor salubrinal was purchased from Calbiochem (La Jolla, CA). Caspase-8 inhibitor Z-IETD-FMK was purchased from BioVision (Mountain View, CA).

### Cell Culture

MDA-MB-231 estrogen-receptor negative human breast cancer cells were purchased from the American Type Culture Collection (Manassas, VA). MCF-7 estrogen-responsive human breast cancer cells were originally provided by Dr. Suzanne Fuqua (Baylor College of Medicine, Houston, TX). Both cell lines were cultured in MEM media with 10% FBS. For experiments, FBS was reduced to 2% and cells were allowed to attach overnight before treatments. α-TEA (40 mM) was dissolved in ethanol as stock solution. Equivalent level of ethanol (0.1%) was used as vehicle control (VEH) for α-TEA treatment (40 µM).

### Quantification of apoptosis

Apoptosis was quantified by Annexin V-FITC/PI assay following the manufacturer's instructions (Invitrogen).

### Western Blot Analyses

Whole cell protein lysates were prepared and western blot analyses were conducted as described previously [24]. Antibodies to the following proteins were used: poly (ADP-ribose) polymerase (PARP), c-FLIP, CHOP, GRP-78, Bcl-2, total JNK, TRAIL and phospho-JNK (pJNK) (Santa Cruz Biotechnology, Santa Cruz, CA) and Bid (Pharmigen, Rockville, MD), phospho-eIF-2α (peIF-2α), eIF-2α (eIF-2α), caspase-8, caspase-9, DR5 and glyceraldehyde-3-phosphate dehydrogenase (GAPDH) (Cell Signaling Technology, Beverly, MA).

### RT-PCR detection of DR5, Bcl-2 and XBP-1 mRNA expression

Total RNA was extracted using RNA isolation kit (Qiagen Inc. Valencia, CA). Semi-quantitative analyses were conducted to detect DR5, Bcl-2 and XBP-1mRNA form by reverse transcriptase-polymerase chain reaction (RT-PCR) using the housekeeping gene β-actin as control. 5 µg total RNA was reverse transcribed to cDNA using 1 µl Superscript RTase (250 U, Invitrogen) following the manufacture's instructions. 1 µl cDNA was used per PCR reaction with 15 µl Taq PCR Master Mix Kit (Qiagen Inc) plus 10 µM oligonucleotide primer pairs (Invitrogen). Primer sequences are available upon request.

### RNA Interference

A scrambled RNA duplex purchased from Ambion (Austin, TX) that does not target any of the known genes was used as the nonspecific negative control for RNAi (referred to as control siRNA). Transfection of MCF-7 or MDA-MB-231 cells with siRNA to DR5, TRAIL, CHOP, JNK, Itch or control (Ambion, Austin, TX) was performed in 100 mm cell culture dishes at a density of 2×10^6^ cells/dish using Lipofectamine 2000 (Invitrogen) and siRNA duplex, resulting in a final siRNA concentration of 30 nM following the company's instructions. After one day exposure to transfection conditions, the cells were re-cultured in 100 mm dish at 2×10^6^ cells/dish and incubated for one day followed by treatments.

### Chromatin immunoprecipiation (ChIP) assay

MDA-MB-231 cells were treated with 40 µM of α-TEA or vehicle (0.1% ethanol) for 15 hours. Protein to DNA cross-linking was conducted by adding 1% formaldehyde to the cell culture medium for 12 min followed by the addition of glycine (0.125M) to stop the cross-linking. Cells were collected for ChIP assay as described by Nelson *et al.* [25]. The CHOP antibody used for the Western blot analysis was used for immunoprecipitation. Normal mouse IgG_1_ purchased from Santa Cruz Biotechnology was used as an isotype control. Polymerase chain reaction (PCR) was conducted using the primers as described by Adbelrahim *et al.* [26] to detect CHOP binding sites in the DR5 promoter region.

### Ectopic Expression of c-FLIP (L)

Transient transfection of MCF-7 cells with wildtype His-tagged FLIP expression construct, pcDNA3.1-His-c-FLIP (L), which was kindly provided by Dr. John C. Reed (The Burnham Inst. La Jolla, CA) [27] was performed following the procedure published before [23]. Transfected cells were cultured overnight before α-TEA treatment.

### Statistical Analyses

Apoptosis data were analyzed using a two-tailed student *t*-test to determine statistical differences among treatments. Differences were considered statistically significant at *p<0.05*.

## Results

### α-TEA induces TRAIL/DR5 and CHOP dependent apoptosis

**Figure 1:**
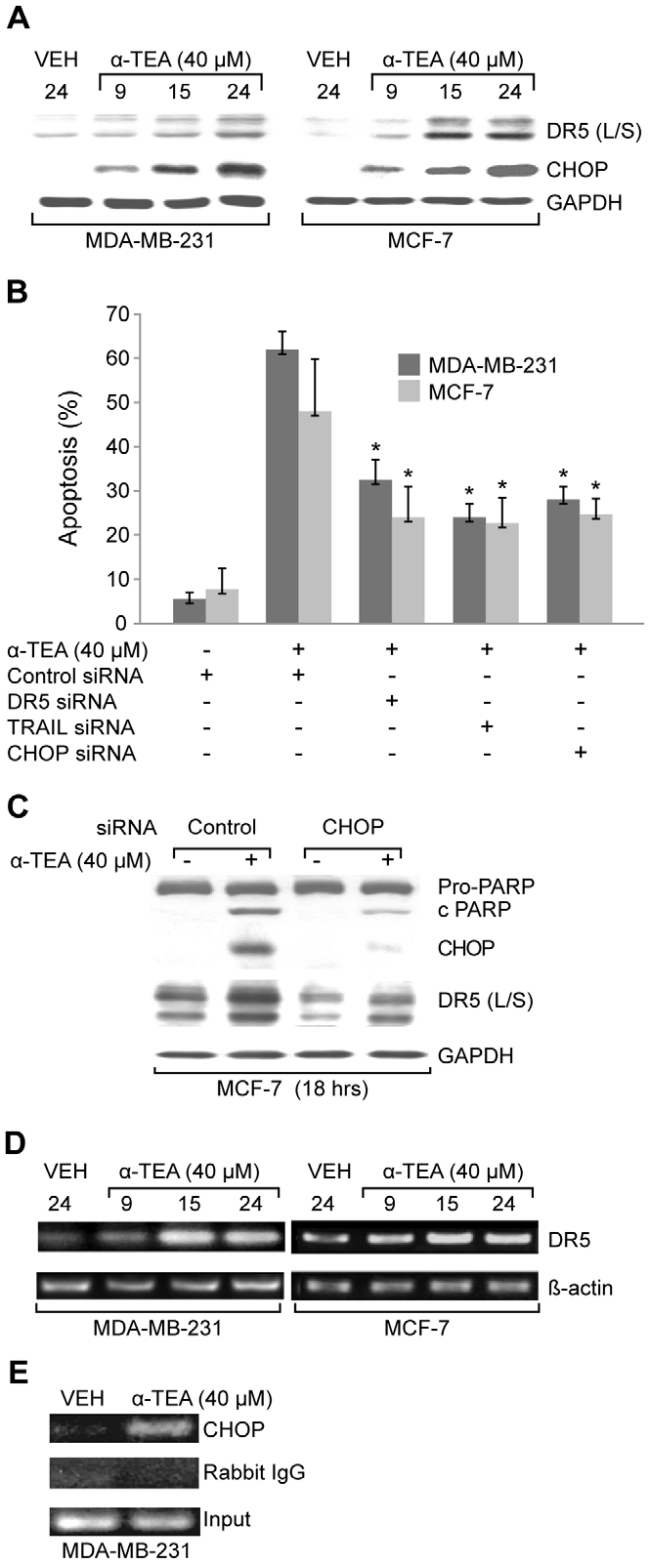
α-TEA induces TRAIL/DR5 and CHOP dependent apoptosis. A. MDA-MB-231 and MCF-7 cells were treated with 40 µM α-TEA for 9, 15, and 24 hrs. Western blot analyses were performed to evaluate DR5 (L/S) and CHOP protein levels using GAPDH as loading control. B. MDA-MB-231 and MCF-7 cells were transiently transfected with siRNAs to DR5, TRAIL or CHOP using non-specific siRNA as negative control followed by treatment with 40 µM α-TEA for 18 hrs. Apoptosis was determined by Annexin V/FACS. C. Samples (from B) were analyzed by western blot for PARP cleavage, CHOP and DR5 (L/S) protein levels using GAPDH as loading control. D. mRNA levels of DR5 were determined by RT-PCR. E. The binding activity of CHOP on DR5 promoter was determined by CHIP assay. Data from A, C, D and E are representative of two or more independent experiments. Data from B are the mean ± S.D. of three independent experiments. * p<0.05 = significantly different from control siRNA determined by *t*-test.

α-TEA treatment of MDA-MB-231 and MCF-7 breast cancer cells up-regulates DR5 and CHOP protein levels in a time dependent manner (Fig 1 A). siRNA knockdown of TRAIL, DR5 or CHOP significantly reduces the ability of α-TEA to induce apoptosis in both cell lines as detected by FACS analyses of expression of the apoptotic biomarker, Annexin V (Fig 1 B), suggesting that both TRAIL/DR5 and CHOP are involved in α-TEA induced apoptosis. Since CHOP has been shown to act as a positive transcriptional factor for DR5 [28], siRNA to CHOP was used to examine if CHOP regulate α-TEA induced upregulation of DR5. Data show that siRNA to CHOP reduced the ability of α-TEA to increase CHOP and DR5 (L/S) levels, as well as to cleave PARP, a biomarker of apoptosis, in MCF-7 cells (Fig 1C). To further confirm that DR5 is regulated by CHOP in α-TEA induced apoptosis RT-PCR to detect mRNA levels of DR5 and ChIP analyses to assess CHOP binding activity on the DR5 promoter were conducted in MDA-MB-231 cells. Data show that α-TEA induces increased levels of DR5 mRNA (Fig 1D) and increased CHOP binding activity on DR5 promoter (Fig 1E) in comparison to VEH treated control cells. These data indicate that CHOP contributes to α-TEA mediated increases in DR5 protein levels and induction of apoptosis.

### α-TEA mediated increases in DR5 and CHOP protein levels are JNK dependent and CHOP influences JNK phosphorylation status

**Figure 2:**
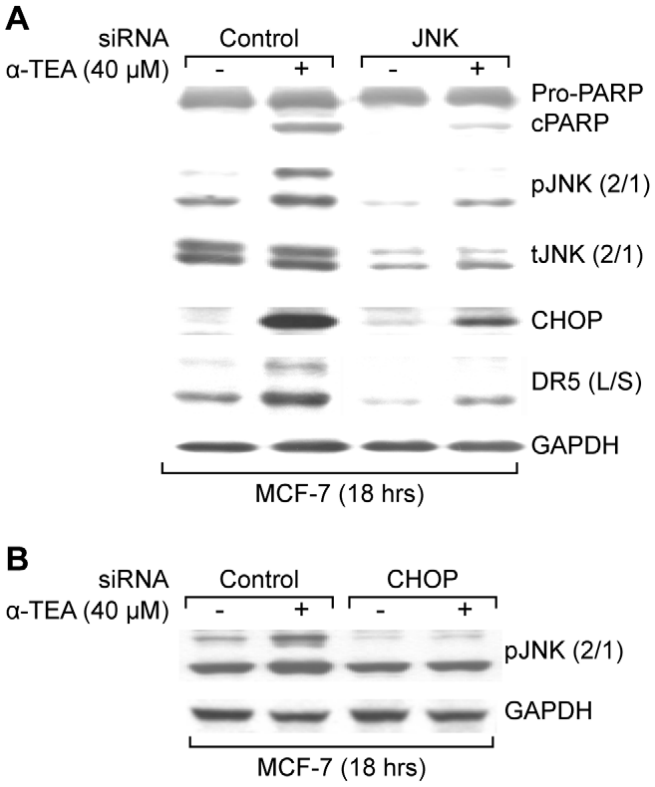
α-TEA induced increased CHOP and DR5 (L/S) protein levels are JNK dependent. In turn, CHOP regulates pJNK2/1 protein levels. A. MCF-7 cells were transiently transfected with siRNA to JNK using non-specific siRNA as negative control followed by treatment with 40 µM α-TEA for 18 hrs. Western blot analyses were performed to evaluate PARP cleavage, pJNK2/1, total JNK2/1, CHOP, and DR5 (L/S) protein levels using GAPDH as loading control. B. MCF-7 cells were transiently transfected with siRNA to CHOP using non-specific siRNA as negative control followed by treatment with 40 µM α-TEA for 18 hrs. Western blot analyses were performed to evaluate pJNK2/1 protein levels using GAPDH as loading control. Data from A and B are representative of two or more independent experiments.

Since prior studies showed that JNK activation is a critical component in α-TEA induced apoptosis and other studies have shown that JNK is involved in upregulation of CHOP protein expression [29] the possible involvement of JNK in α-TEA mediated increases in CHOP/DR5 protein expression was investigated in MCF-7 cells. siRNA to JNK reduced total JNK and reduced the ability of α-TEA to increase phosphorylated JNK2/1 (pJNK), increase CHOP and DR5 (L/S) protein levels and to induce apoptosis as measured by PARP cleavage (Fig 2A), suggesting that JNK is a critical contributor to increases in CHOP and DR5 (L/S) protein level. Interestingly, siRNA to CHOP reduced α-TEA mediated increases in pJNK2/1 protein levels (Fig 2B), suggesting that α-TEA mediated increases in JNK phosphorylation are dependent on CHOP expression.

### Endoplasmic reticulum stress involvement in α-TEA induced apoptosis contributes to JNK/CHOP/DR5 upregulation

**Figure 3:**
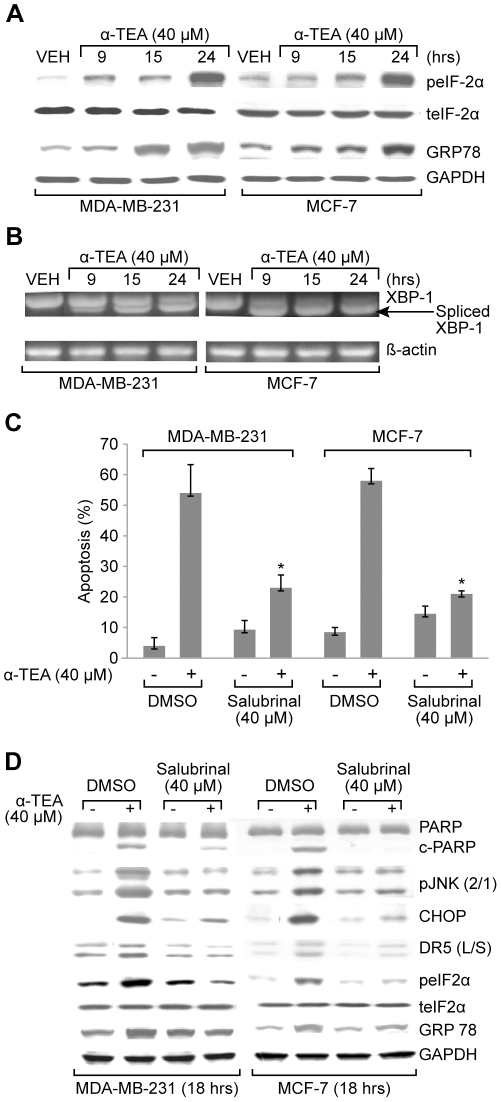
ER stress is involved in α-TEA induced apoptosis and contributes to JNK/CHOP/DR5 upregulation. A. MDA-MB-231 and MCF-7 cells were treated with 40 µM α-TEA for 9, 15, and 24 hrs. Western blot analyses were performed to determine ER stress markers; peIF-2α, total eIF2α and GRP78 protein levels. GAPDH levels were used as lane controls. B. Splicing of XBP-1, a marker for ER stress, was determined by RT-PCR. C. Both cell lines were treated with ER-stress inhibitor salubrinal at 40 µM plus 40 µM α-TEA for 18 hrs. Apoptosis was determined by Annexin V/FACS. D. Western blotting was conducted to evaluate salubrinal effects on α-TEA induced cleavage of PARP, upregulation of pJNK/2/1, CHOP, DR5 (L/S), peIF2α, total eIF2α, and GRP78 proteins. Data from A, B and D are representative of at least 2 individual experiments. Data from C are presented as the mean ± S.D. of three independent experiments. * *P*<0.05 =  ER-stress inhibitor salubrinal at 40 µM plus 40 µM α-TEA treatment is significantly different from 40 µM α-TEA determined by *t*-test.

α-TEA treatment of MDA-MB-231 and MCF-7 cells increased levels of endoplasmic reticulum stress (ER-stress) indicators: phosphorylated eukaryotic initiation factor 2-alpha (peIF-2α) and glucose-regulated protein of 78kDA (GRP78) also known as BiP (Fig 3A), as well as spliced mRNA forms of X-box binding protein-1 (XBP-1) (Fig 3B) in a time-dependent manner, suggesting that α-TEA treatment induced ER stress in both cell lines. Furthermore, ER-stress inhibitor salubrinal significantly reduced the ability of α-TEA to induce apoptosis detected by Annexin V/FACS (Fig 3C). Since JNK/CHOP/DR5 pathway can be activated in response to ER stress [30], studies were conducted to examine if α-TEA mediated JNK/CHOP/DR5 (L/S) signaling events via ER stress. Data show that ER-stress inhibitor salubrinal significantly reduced the ability of α-TEA to upregulate pJNK2/1, CHOP, and DR5 (L/S), as well as to induce PARP cleavage in both cell lines (Fig. 3D). Salubrinal significantly reduced the ability of α-TEA to upregulate ER stress markers GRP78 and peIF2α, confirming the efficiency of ER-stress inhibitor. These data demonstrate that α-TEA induction of JNK/CHOP/DR5 and apoptosis is mediated via ER stress.

### α-TEA induction of ER stress-dependent JNK/CHOP/DR5 signaling is mediated by TRAIL/DR5 pathway

**Figure 4:**
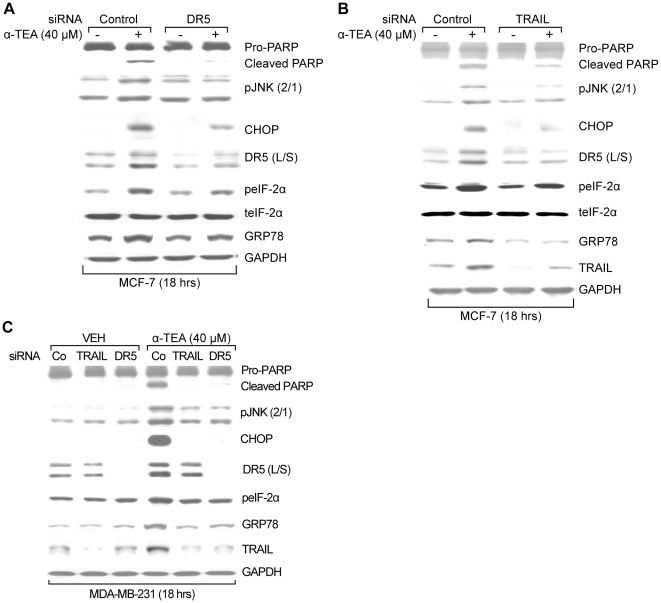
ER stress dependent JNK/CHOP/DR5 upregulation is mediated by TRAIL/DR5 pathway in α-TEA induced apoptosis. A & B. MCF-7 cells were transiently transfected with siRNA to DR5 or TRAIL, using non-specific siRNA as negative control followed by treatment with 40 µM α-TEA for 18 hrs. Western blot analyses were performed to evaluate PARP cleavage, pJNK2/1, CHOP, DR5 (L/S), peIF-2α total eIF-2α and GRP78 protein levels, using GAPDH as loading control for (A & B) and TRAIL protein levels for siRNA knockdown efficiency (B). C. MDA-MB-231 cells were transiently transfected with siRNA to DR5 or TRAIL, using non-specific siRNA as negative controls followed by treatment with 40 µM α-TEA for 18 hrs. Western blot analyses were performed to evaluate PARP cleavage, pJNK (2/1), CHOP, DR5 (L/S), peIF-2α, GRP78, and TRAIL protein levels, using GAPDH as loading control. Data from A, B and C are representative of at least 2 individual experiments.

Since both JNK and DR5 can be regulated by TRAIL/DR5 signaling [30], [31], studies were conducted to determine if α-TEA mediated ER stress events are downstream events of TRAIL/DR5 using siRNA knockdown procedures. MCF-7 and MDA-MB-231 cells transiently transfected with siRNAs to DR5 or TRAIL exhibited reduced levels of DR5 and TRAIL, respectively, and showed blockage of α-TEA's ability to produce PARP cleavage and to increase levels of pJNK2/1, CHOP, DR5 (L/S), as well as blocked α-TEA's ability to upregulate ER stress markers GRP78 and peIF-2α (Fig 4 A, B & C), demonstrating that α-TEA induction of ER stress-dependent JNK/CHOP/DR5 signaling is mediated by TRAIL/DR5 pathway.

### ER stress dependent JNK/CHOP/DR5 upregulation occurs both upstream and downstream of caspase-8 activation

**Figure 5:**
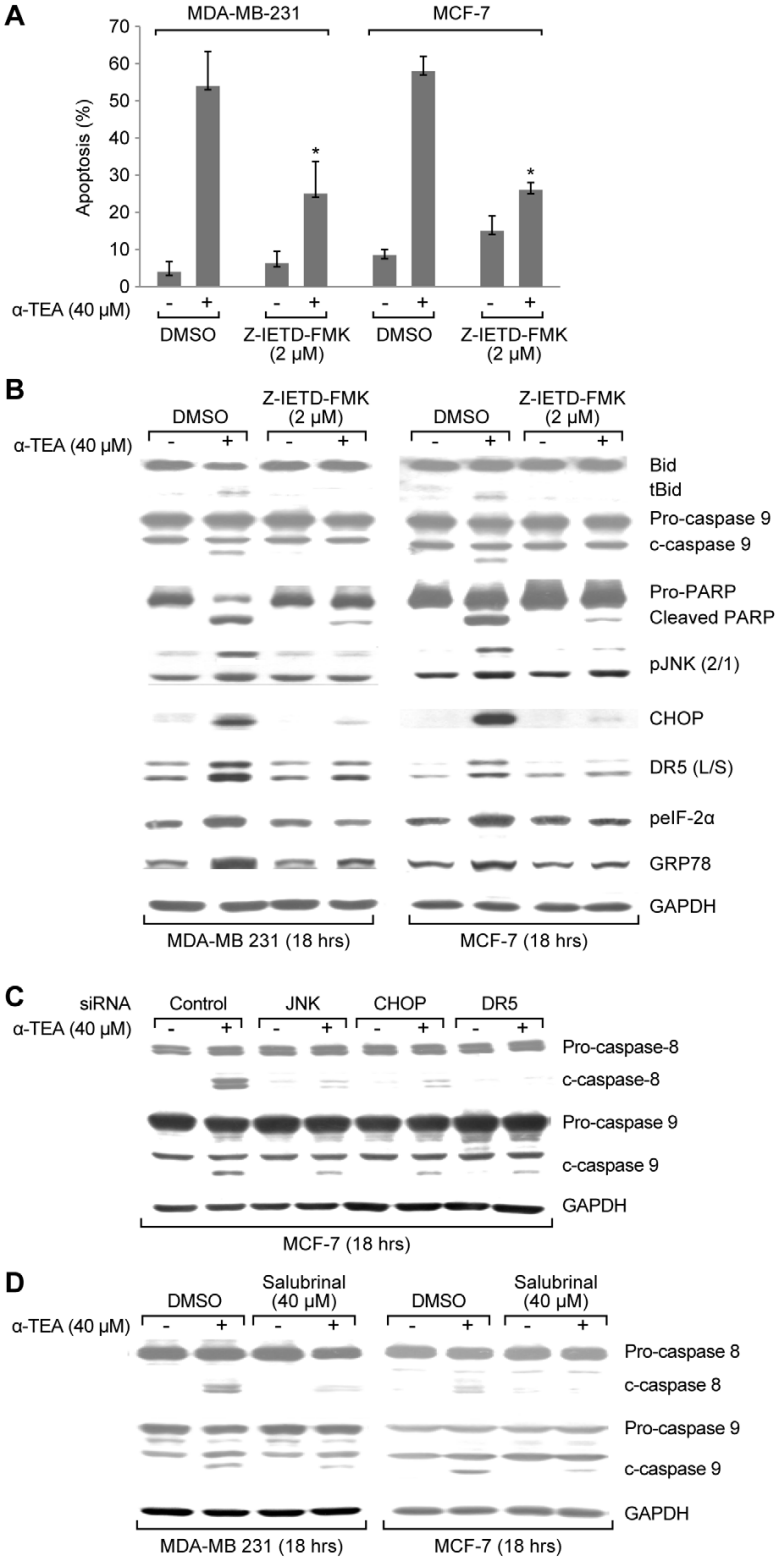
ER stress-dependent JNK/CHOP/DR5 upregulation is both upstream and downstream events of caspase-8. A. MDA-MB-231 and MCF-7 cells were cultured with caspase-8 inhibitor (Z-IETD-FMK) or DMSO vehicle control plus 40 µM α-TEA for 18 hrs. Apoptosis was determined by Annexin V/FACS. B. Samples (from A) were analyzed by western blot to evaluate caspase-8 cleavage of Bid to tBid, cleaved caspase 9 and PARP, pJNK2/1, CHOP, DR5 (L/S), peIF-2α and GRP78 protein levels using GAPDH as loading control. C. MCF-7 cells were transiently transfected with siRNA to JNK, CHOP and DR5, using non-specific siRNA as negative control followed by treatment with 40 µM α-TEA for 18 hrs. Western blot analyses were performed to evaluate caspase-8 and -9 cleavages, GAPDH served as lane controls. D. MDA-MB-231 and MCF-7 cells were cultured with ER-stress inhibitor salubrinal at 40 µM plus 40 µM α-TEA for 18 hrs. Western blot was conducted to evaluate caspase-8 and -9 cleavages, GAPDH served as lane controls. Data from B, C and D are representative of two or more individual experiments. Data from A are presented as the mean ± S.D. of three independent experiments. * *P*<0.05 =  caspase-8 inhibitor (Z-IETD-FMK) at 2 µM plus 40 µM α-TEA treatment is significantly different from 40 µM α-TEA determined by *t*-test.

In an effort to determine if ER stress proceeds or follows α-TEA activation of DR5 signaling, caspase-8 inhibitor (Z-IETD-FMK) was employed. Data show that caspase-8 inhibitor significantly reduced α-TEA abilities to induce apoptosis (Fig 5A), and cleavage of Bid, caspase-9 and PARP, as well as increase protein levels of pJNK2/1, CHOP, DR5, and ER stress markers GRP78 and peIF-2α in both cell types (Fig 5B). These data suggest that α-TEA activation of caspase-8 is upstream of ER-stress mediated JNK/CHOP/DR5 signaling. siRNA knockdown of JNK, CHOP and DR5 in MCF-7 cells reduced the ability of α-TEA to cleave caspases 8 and 9 (Fig 5 C), suggesting that not only DR5 but also JNK and CHOP are involved in activation of caspases-8 and -9. Furthermore, treatment of MDA-MB-231 and MCF-7 cells with ER inhibitor salubrinal blocked α-TEA's ability to induce caspases-8 and -9 cleavage (Fig 5 D), providing additional evidence for caspase 8 acting prior to and after α-TEA induced ER stress.

### α-TEA down regulation of c-FLIP (L) protein levels is in part, mediated by caspase-8/ER stress/JNK/CHOP/DR5 signaling via Itch E3 ligase ubiquitination

**Figure 6:**
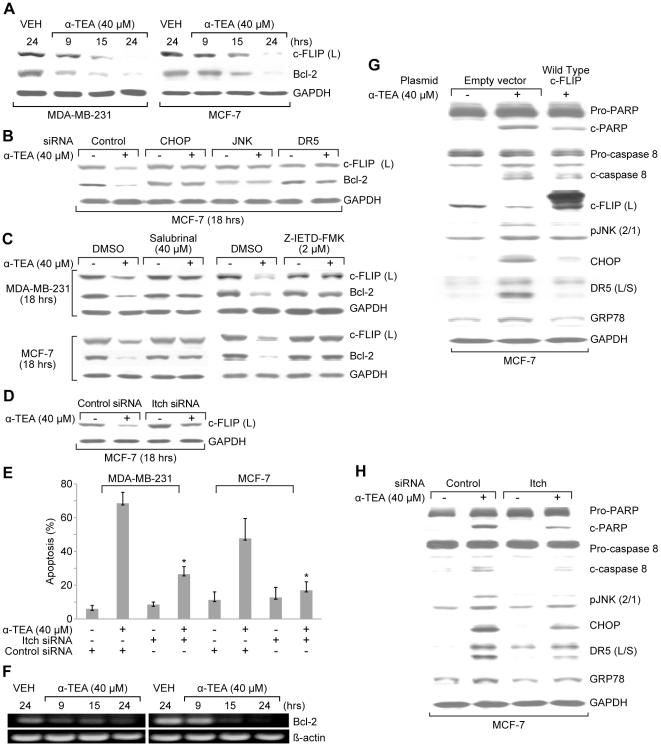
α-TEA decreased Bcl-2 and c-FLIP (L) protein levels via caspase-8 dependent ER stress-mediated JNK/CHOP/DR5 pathway. A. MDA-MB-231 and MCF-7 cells were treated with 40 µM α-TEA for 9, 15, and 24 hrs. Western blot analyses were performed to evaluate Bcl-2 and c-FLIP (L) protein levels with GADPH as loading control. B. MCF-7 cells were transiently transfected with JNK, CHOP, and DR5 siRNA or control siRNA followed by treatment with 40 µM α-TEA or vehicle for 18 hrs. Bcl-2 and c-FLIP (L) protein levels were determined by western blot analyses, using GAPDH as loading control. C. MDA-MB-231 and MCF-7 cells were cultured with ER-stress inhibitor salubrinal at 40 µM or caspase-8 inhibitor (Z-IETD-FMK) at 2 µM plus 40 µM α-TEA for 18 hrs. Bcl-2 and c-FLIP (L) protein levels were determined by western blot analyses, using GAPDH as loading control. D. MCF-7 cells were transiently transfected with Itch siRNA or control siRNA followed by treatment with 40 µM α-TEA or vehicle for 18 hrs. c-FLIP (L) protein levels were determined by western blot analyses. E. MDA-MB-231 and MCF-7 cells were transiently transfected with siRNA to itch using non-specific siRNA as negative control followed by treatment with 40 µM α-TEA for 18 hrs. Apoptosis was determined by Annexin V/FACS. F. MDA-MB-231 and MCF-7 cells were treated with 40 µM α-TEA for 9, 15, and 24 hrs. mRNA levels of Bcl-2 were determined by RT-PCR G. MCF-7 cells were transiently transfected with wild-type c-FLIP plasmid or vector control followed by treatment with 40 µM α-TEA for 18 hrs. Western blot analyses were performed to evaluate over expression of c-FLIP on ability of α-TEA to cleave PARP and caspase-8, and down-regulate c-FLIP (L), pJNK2/1, CHOP, DR5 (L/S), peIF-2α and GRP78 protein expression using GAPDH as loading control. H. MCF-7 cells were transiently transfected with siRNA to Itch, using non-specific siRNA as negative control followed by treatment with 40 µM α-TEA for 18 hrs. Western blot analyses were performed to evaluate elevated levels of c-FLIP (L) on ability of α-TEA to cleave PARP and caspase-8 and to down-regulate pJNK2/1, CHOP, DR5 (L/S) and GRP78 protein levels using GAPDH as loading control. Data for A, B, C, D, F, G and H are representative of two or more individual experiments. Data from E are the mean ± S.D. of three independent experiments. * p<0.05 = significantly different from control siRNA determined by *t*-test.

α-TEA downregulates c-FLIP (L) protein levels in both MDA-MB-231 and MCF-7 breast cancer cells in a time dependent manner (Fig 6A). MCF-7 cells transiently transfected with siRNA to CHOP, JNK or DR5, and both MDA-MB-231 and MCF-7 cells cultured with ER-stress inhibitor salubrinal or caspase 8 inhibitor Z-IETD-FMK prevented the ability of α-TEA to decrease c-FLIP (L) protein levels (Fig 6B and C), providing evidence that α-TEA downregulation of c-FLIP is mediated by caspase-8/ER stress/JNK/CHOP/DR5 signaling. Since c-FLIP has been reported to be degraded via JNK-dependent E3 ubiquitin ligase Itch [32], studies were conducted to determine if Itch E3 ligase mediated ubiquitination was involved in α-TEA down-regulation of c-FLIP. Knockdown of Itch E3 ligase with siRNA to Itch reduced α-TEA's ability to downregulate c-FLIP (L) protein levels in MCF-7 cells (Fig. 6D). siRNA to Itch significantly reduced the ability of α-TEA to induce apoptosis in MDA-MB-231 and MCF-7 cells (Fig 6E). These data suggest that α-TEA induced downregulation of c-FLIP (L) is mediated by a caspase-8/ER stress/JNK/CHOP/DR5 pathway via Itch ligase ubiquitination.

### α-TEA downregulation of c-FLIP mediates ER stress-dependent JNK/CHOP/DR5 signaling via activation of caspase-8

Since c-FLIP is an inhibitor of caspase-8 activation, we hypothesized that α-TEA downregulation of c-FLIP may contribute to activation of caspase-8 and subsequent ER stress-dependent JNK/CHOP/DR5 signaling. To test this hypothesis, MCF-7 cells were transiently transfected with wildtype c-FLIP (L) plasmid or Itch siRNA followed by treatment with α-TEA (40 µM) for 18 hrs. Over-expression of c-FLIP enhanced c-FLIP (L) levels (Fig 6G) and siRNA to itch blocked α-TEA's ability to reduce c-FLIP protein expression (Fig 6D). Both overexpression of c-FLIP (L) protein and restoration of c-FLIP protein expression by silencing itch in α-TEA treatment reduced the ability of α-TEA to cleave PARP and caspase-8, and to increase protein levels of pJNK2/1, CHOP, DR5, and GRP 78 (Fig 6G and H). These data suggest that α-TEA's ability to downregulate c-FLIP (L) is important for caspase-8 activation and subsequent activation of the ER stress/JNK/CHOP/DR5 signaling.

### α-TEA downregulation of Bcl-2 protein and mRNA expression is mediated by ER stress

α-TEA treatment of MDA-MB-231 and MCF-7 cells reduced both Bcl-2 protein and mRNA levels in a time dependent manner (Fig 6A and F). siRNA to JNK, CHOP, and DR5 (Fig 6B) as well as caspase-8 and ER stress inhibitors (Fig 6C) blocked the ability of α-TEA to decrease Bcl-2 protein expression. These data suggest that α-TEA induced down-regulation of Bcl-2 is mediated by caspase-8, ER stress, JNK, CHOP and DR5.

## Discussion

**Figure 7:**
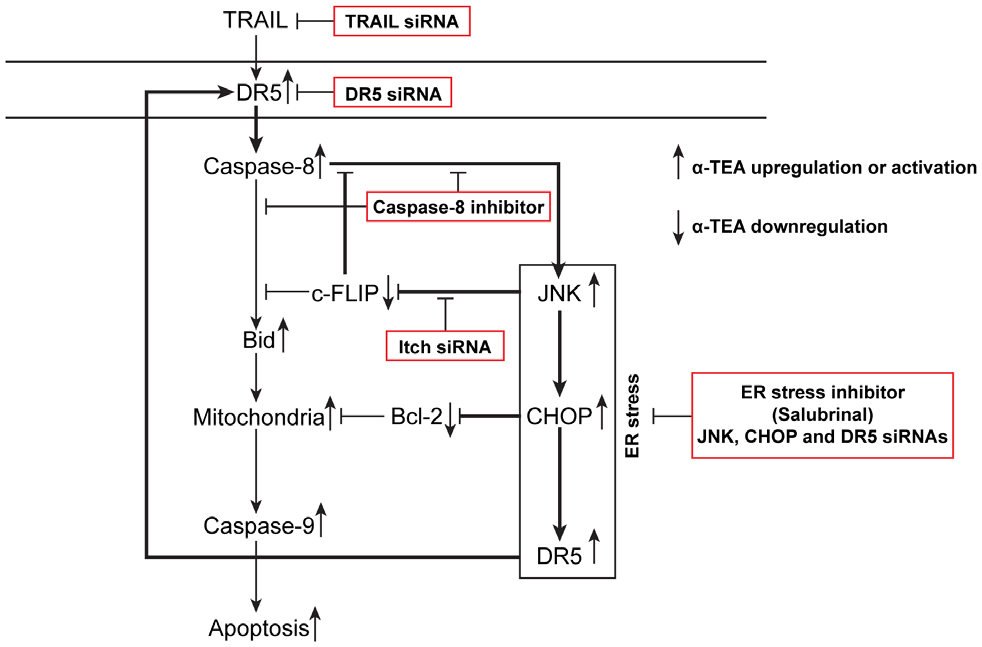
Schematic diagram showing α-TEA to induce breast cancer cells to undergo apoptosis via caspase-8 mediated ER stress JNK/CHOP/DR5 amplification loop and by down-regulation of anti-apoptotic mediators c-FLIP (L) and Bcl-2.

The underlying mechanisms of how α-TEA, a potential chemotherapeutic drug, enhances DR5 death signaling pathways were investigated. For the first time, studies demonstrate that: (i) α-TEA induces ER stress dependent apoptosis, (ii) α-TEA induced ER stress regulates JNK/CHOP/DR5 signaling and is mediated by TRAIL/DR5/caspase-8, forming a positive loop, (iii) α-TEA downregulation of c-FLIP is mediated by ER stress-dependent JNK/CHOP/DR5 signaling via JNK activation of Itch E3 ligase ubiquitination and involved in activation of the ER-stress-dependent events via reducing the inhibitory effect of c-FLIP on caspase-8, and (iv) α-TEA downregulation of Bcl-2 protein levels is mediated by the ER stress-dependent events. Taken together, these data show that α-TEA induces TRAIL/DR5/caspase-8 dependent ER stress that triggers JNK/CHOP/DR5 signaling enhancing DR5/caspase-8 mediated mitochondria-dependent apoptotic signaling and produces a downregulation of two key anti-apoptotic factors; c-FLIP and Bcl-2. Based on these data, we provide a schematic diagram depicting a series of events including a positive-acting feedback loop, which is operative in α-TEA induced DR5 dependent apoptosis of human breast cancer cells in culture (Fig 7). DR5 signaling triggers caspase-8 activation, leading to two α-TEA initiated pathways: (i) a classic mitochondria-dependent apoptotic cascade initiated by cleavage of Bid and (ii) an ER-stress dependent activation of JNK/CHOP/DR5 sequence leading to further activation of caspase-8 and downregulation of anti-apoptotic factors; c-FLIP and Bcl-2. Importantly, down-regulation of c-FLIP serves to further enhance caspase-8 activation, which sustains the ER-stress/JNK/CHOP/DR5 amplifying loop.

TRAIL has emerged as a potent anticancer agent based on its ability to induce apoptosis in tumor cells but not in most normal cells [1], [2], [3]. Thus, targeting TRAIL/DR4/DR5 signaling pathways holds promise for inducing pro-apoptotic signaling in many cancer types while sparing normal cells and tissues [33], [34]. Although considerable effort has been made in investigating the biological ramifications of TRAIL death receptor signaling, a complete understanding of the multiple pathways and events involved remain unclear. Typically, signaling pathways that initiate apoptosis have been broadly classified as (i) extrinsic death receptor initiated pathways or (ii) intrinsic pathways initiated by mitochondrial events [35]. These two pathways are not mutually exclusive, and JNK and caspase-8 have been observed to play central roles in both [4], [36]. Our findings that TRAIL/DR5/casapse-8 triggers an ER stress dependent JNK/CHOP/DR5 amplifying loop and a JNK/c-FLIP/caspase-8 amplifying loop in α-TEA induced apoptosis provides new insights into TRAIL/DR5-mediated apoptotic signaling.

DR5 upregulation has been reported to be mediated by NF-kB in TRAIL- induced apoptosis in MCF-7 and MDA-MB-231 cells [37]. However, inhibition of NF-kB using IkB phosphorylation inhibitor BAY-11-7085 [38] enhances α-TEA induced upregulation of DR5 and apoptosis in MCF-7 cells (data not shown), suggesting that NF-kB plays an anti-apoptotic role in α-TEA-induced apoptosis. Data reported here showing that α-TEA up-regulates DR5 via CHOP binding to the DR5 promoter is supported by published reports showing that CHOP can directly regulate DR5 expression through a CHOP binding site in the 5′-flanking region of the DR5 promoter [28], [39].

JNK is a stress responsive kinase that has been reported to induce CHOP expression via an AP-1 binding site in the CHOP promoter [40], [41]. JNK has been shown to be activated and is required for α-TEA-induced apoptosis of human breast, ovarian and prostate cancer cells [17], [18], [19], [20]. Data presented here show that siRNA to JNK blocks α-TEA induced CHOP as well as DR5, indicating that CHOP/DR5 upregulation is mediated via JNK in α-TEA induced apoptosis. These data are in agreement with other studies showing that CHOP is an ER stress-regulated protein [42], JNK activation can contribute to CHOP expression during ER stress [43], and coupling of JNK/CHOP/DR5 are involved in ER stress-mediated apoptosis [29].

Present study show that α-TEA induces increased levels of peIF-2α and GRP78 proteins and spliced XBP-1, all of which are recognized as ER stress markers [44], [45] and that salubrinal, a selective inhibitor of eIF-2α dephosphorylation (protects cells from ER stress) [46] blocked α-TEA induced apoptosis and the ability of α-TEA to activate JNK/CHOP/DR5 and upregulate ER stress markers, confirming that ER stress is induced by α-TEA and is involved in both apoptosis and activation of JNK/CHOP/DR5.

Present data show that α-TEA induction of the ER stress dependent JNK/CHOP/DR5 pathway is a downstream consequence of TRAIL/DR5 signaling. Furthermore, a caspase-8 inhibitor not only blocks α-TEA induced mitochondria-dependent apoptotic cascade as indicated by blockage of Bid and pro-caspase-9 cleavage, but also blocks ER stress signaling events, indicating that TRAIL/DR5 downstream effector caspase-8 contributes to α-TEA induced ER stress-dependent events. To our knowledge, this is the first report that ER stress can be triggered by a TRAIL/DR5/caspase-8 pathway and that ER stress induces a JNK/CHOP/DR5 amplifying loop that can further enhance TRAIL/DR5-mediated apoptosis. The mechanism(s) whereby activated caspase-8 induces ER stress were not investigated in this study. However, studies using hepatoblastoma HepG2 cells suggest that activated caspase-8 may promote and amplify the ER stress response by cleaving BAP31, an integral membrane protein of the ER, in glycochenodeoxycholic acid-induced apoptosis [47].

As a caspase-8 inhibitor, c-FLIP plays an important role in death receptor dependent apoptosis. Published data showed that down-regulation of c-FLIP is involved in α-TEA induced apoptosis in human ovarian and prostate cancer cells [18], [22], [23]. However, how α-TEA regulates c-FLIP is not known. Data reported here show that down-regulation of c-FLIP is mediated by the caspase-8 dependent ER stress JNK/CHOP/DR5 loop via JNK-mediated phosphorylation and activation of the E3 ubiquitin ligase Itch. JNK-mediated protein degradation of c-FLIP has been reported for TNF-induced cell death [48]. However, it is the first report that JNK-mediated c-FLIP degradation is ER stress dependent.

Bcl-2 is a well established anti-apoptotic factor, which inhibits mitochondrial dependent apoptosis. It has been reported that ER stress induces downregulation of Bcl-2 via CHOP [49]. Although CHOP is a transcription factor, a CHOP responsive site in the Bcl-2 promoter has not been reported; suggesting that if CHOP mediates transcriptional regulation of Bcl-2 expression it must form a complex with other proteins [49]. Here, for the first time, we demonstrate that α-TEA downregulates Bcl-2 at both protein and mRNA levels, which is regulated by ER-stress mediated JNK/CHOP/DR5 signaling.

In conclusion, data show that α-TEA induces a TRAIL/DR5/caspase-8 dependent ER stress-mediated JNK/CHOP/DR5 loop, which amplifies caspase-8-mediated mitochondrial-dependent apoptosis via downregulation of c-FLIP and Bcl-2. These data are significant in that they provide a better understanding of the pro-apoptotic actions of the anti-cancer agent α-TEA, and provide new insights into DR5-mediated apoptotic signaling in human breast cancer cells.
